# Comparison of echocardiographic and cardiac magnetic resonance imaging measurements of systolic function during breast cancer therapy

**DOI:** 10.1186/s12872-024-04262-7

**Published:** 2025-09-15

**Authors:** Judith Huynh, Andreas Malmgren, Morten Kraen, Elin Trägårdh, Magnus Dencker

**Affiliations:** 1 Department of Medical Imaging and Physiology, Skåne University Hospital, Malmö, Sweden; 2 Department of Translational Medicine, Lund University, Malmö, Sweden

**Keywords:** Cardiotoxicity, Global longitudinal strain, Mitral annular plane systolic excursion

## Abstract

**Background:**

To compare echocardiographic and cardiac magnetic resonance imaging (CMR) measurements of global longitudinal strain (GLS) and global mitral annular plane displacement in women with breast cancer undergoing chemotherapy. The study focused on assessing the mitral annular plane systolic excursion (MAPSE) in echocardiography (ECHO) and atrioventricular plane displacement (AVPD) in CMR as parameters for global mitral annular plane displacement.

**Material and method:**

Consecutive breast cancer patients (*n* = 16) were evaluated with ECHO and CMR before, during and after chemotherapy. Echocardiographic GLS was analyzed using two different software programs (TomTec and QLab). Non-parametric Wilcoxon’s signed-rank test, Bland-Altman plots and Friedman’s test were used for the statistical analyses. A statistical significance level of all analyses was set at a p-value < 0.05. The study was approved by National Ethics Review Board in Sweden (DNR 2019–04588).

**Results:**

No significant differences were found in GLS at baseline between ECHO (median: QLab − 20.4% and TomTec − 22.0%) and CMR (median: -19.5%) (ECHO(QLab) vs. CMR *p* = 0.733 and ECHO(TomTec) vs. CMR *p* = 0.093). After chemotherapy significant reductions in GLS were measured with ECHO(TomTec) (median: -20.1, *p* = 0.035) and CMR (median GLS: -17.4%, *p* = 0.004). At baseline ECHO-MAPSE (median: 16.8 mm) and CMR-AVPD (median: 14.0 mm) differed significantly (*p* = 0.015). However, no significant reduction of MAPSE (median: 15.5 mm) or AVPD (median: 13.8 mm) were detected after chemotherapy (*p* = 0.076 respective *p* = 0.706). Though ECHO-MAPSE showed a tendency to decrease after chemotherapy, CMR-AVPD did not.

**Conclusion:**

ECHO(TomTec)-GLS is as compatible to detect early signs of cardiotoxicity as CMR. ECHO-MAPSE could be more sensitive than CMR-AVPD to detect subtle changes during chemotherapy.

**Supplementary Information:**

The online version contains supplementary material available at 10.1186/s12872-024-04262-7.

## Background

Early detection of cancer therapy-related cardiac dysfunction (CTRCD) is crucial for successful cancer and cardiac treatment [1]. Numerous guidelines recommend regular cardiac monitoring before, during and after ended chemotherapy to enhance the likelihood of detecting early signs of cardiac changes [2–4]. Until now, left ventricle ejection fraction (LVEF) has been the most widely used and strongest predictor for identifying cardiotoxicity. However, recent expert consensuses suggest that LVEF is an insensitive parameter to detect early myocardial changes. Therefore, more sensitive methods or parameters are required to identify earlier stages of myocardial damage [3, 5].

Guidelines have presented global longitudinal strain (GLS) as a suitable echocardiographic parameter to detect early signs of CTRCD [5]. GLS is relatively independent of LVEF and has demonstrated greater sensitivity compared to conventional 2D LVEF [6, 7]. Studies have shown that GLS decreases while 2D LVEF may still be preserved. Moreover, GLS is a more reproducible and accurate measurement of myocardial function compared to conventional LVEF [5, 8, 9].

The longitudinal ventricle function is assumed to be the first factor to be affected in myocardial dysfunction. LVEF may still be preserved since the circumferential function is compensating for the global function. In echocardiography (ECHO), mitral annular plane systolic excursion (MAPSE) is the parameter for assessing mitral annular plane displacement. MAPSE is considered a sensitive and reproducible parameter to detect subtle global longitudinal left ventricle dysfunction [10, 11]. Additionally, MAPSE provides high frame rate (FR), making it particularly sensitive for detecting subtle wall motion changes.

Cardiac magnetic resonance imaging (CMR) is generally regarded as the reference method for evaluating chamber volumes and function. This imaging modality is independent of the acoustic window and geometric assumptions, enabling more accurate and reproducible measurements of the cardiac function than ECHO. Besides providing reliable measurements of the cardiac chambers, newer CMR techniques allow assessments of myocardial strain [12] as well as mitral annular plane displacement, referred to as atrioventricular plane displacement (AVPD) in CMR and is the corresponding parameter to MAPSE in ECHO [13].

The aim of this study was to compare echocardiographic and cardiac magnetic resonance imaging measurements of myocardial dysfunction in breast cancer patients undergoing cardiotoxic cytostatic treatments.

## Material and method

### Study population

**Fig. 1:**
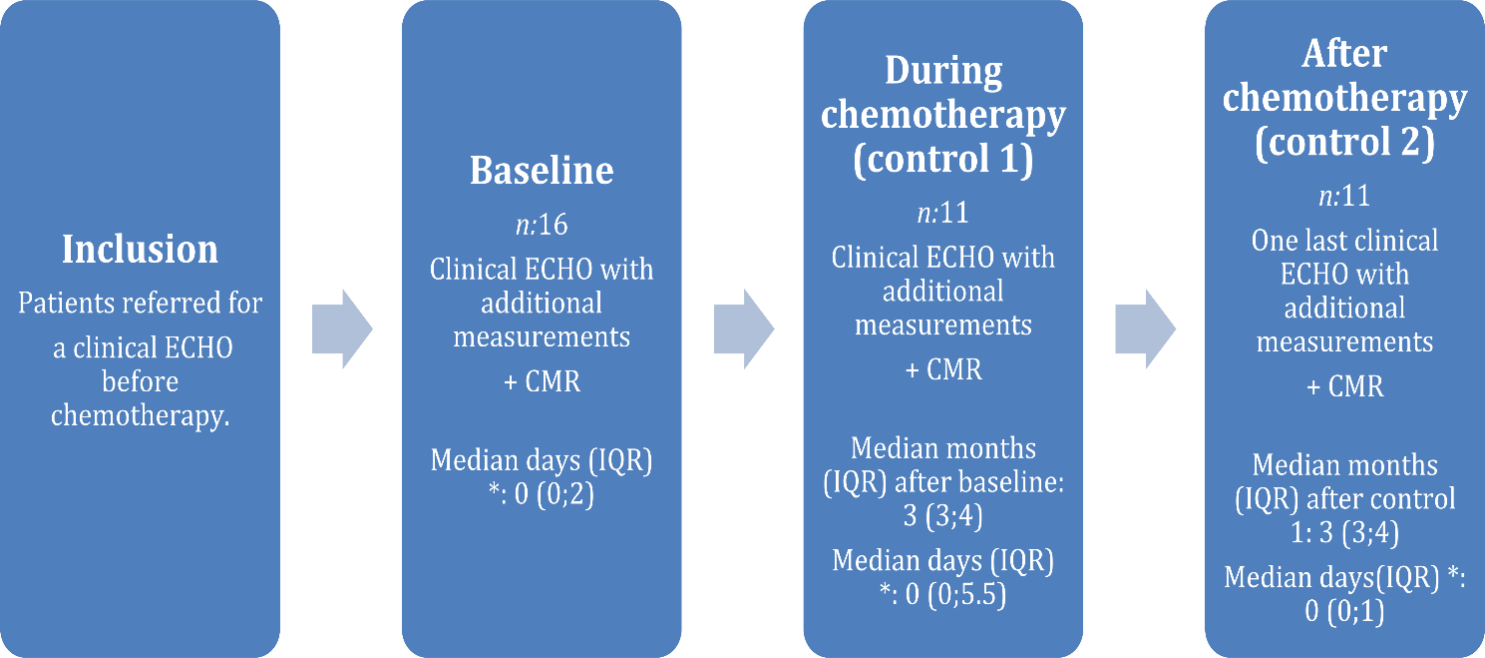
Flowchart of the process of the study. *, between ECHO and corresponding CMR; ECHO, echocardiography; CMR, cardiac magnetic resonance imaging

Consecutive, newly diagnosed breast cancer patients, who received cytostatic treatments, were recruited for this prospective cohort study. Written consent was obtained from all participants. These patients were referred to the Department of Medical Imaging and Physiology at Skåne University Hospital in Malmö by their oncologist for cardiac monitoring with echocardiography before, during and after their breast cancer cytostatic treatment. The exclusion criteria in this study were if the patient had already received cytostatic drugs for their current breast cancer treatment before their first cardiac check-up with ECHO (baseline assessment) or patient with contraindications for CMR. In connection with the ECHO examination, a CMR examination was also performed on the patient as soon as possible before the initiation of cytostatic drugs. Subsequent follow-up examinations were conducted with both ECHO and CMR. The CMR examination was scheduled to occur within 2 weeks of the corresponding ECHO examination. After completion of cytostatic treatment, a final ECHO and CMR examination was performed for this study (Fig. 1). The study was approved by National Ethics Review Board in Sweden (DNR 2019–04588).

### Echocardiography

Transthoracic echocardiography was performed in all participants using the EPIQ 7 ultrasound machine (Philips Healthcare, The Netherlands) equipped with a 1–5 MHz phased array transducer X5-1 (Philips Ultrasound, USA). Still images and cine-loops were acquired in DICOM format and stored in the external platform Intellispace Cardiovascular (ISCV) 2.3 (Philips Medical Systems, The Netherlands).

Sector width and depth were optimized to achieve a FR of > 50 frames per second (fps) in 2D images. All cine-loops consisted of two or more consecutive cardiac cycles. The echocardiographic examinations were performed according to American Society of Echocardiography (ASE) guidelines [14]. Apical 4-,2- and 3-chamber views were obtained from the clinical protocol with a FR > 50 fps to be able to achieve adequate GLS. Apical 4- and 2-chamber views were also used to estimate LVEF Simpson’s biplane and chamber volumes. For the study, MAPSE of the septal, lateral, anterior and inferior walls were obtained from apical 4- and 2-chamber view using M-mode (see additional file S1).

All echocardiographic measurements were post-processed by one experienced observer, who was blinded from the other corresponding measurements. GLS was post-processed offline and evaluated with two different software programs, QLab (Philips Healthcare, The Netherlands) and TomTec (TomTec Imaging Systems, Germany). Both software programs are commonly used for GLS assessment in clinical practice. Their inclusion in this study helps identify potential inter-vendor variability and provide insights into how different software programs may influence the assessment of myocardial strain. The software automatically traced the myocardium of the left ventricle in all three apical chamber views. The operator then confirmed the automatic tracing of the myocardium or made manual adjustments if necessary. After processing all three apical chamber views, the program calculated the average peak systolic GLS for the left ventricle (see additional file S2).

### Cardiac magnetic resonance imaging

All CMR studies were performed on a 1.5 Tesla scanner, Avanto (Siemens Healthcare, Germany). The study protocol consisted of short-axis and long-axis cine imaging acquired with steady-state free precession sequences for evaluation of cardiac volumes, function and strain calculations. Measurements of GLS and AVPD, were performed by a single blinded reader using the software Segment v 3.3 (Medviso AB, Sweden) (see additional file S3 and S4).

### Statistical analysis

IBM SPSS Statistics, Version 28.0 (IBM Corp, USA) software was used for all statistical analyses. The non-parametric Wilcoxon’s signed-rank test was used to compare baseline GLS and MAPSE/AVPD values obtained with ECHO and CMR. Bland-Altman plots were used to illustrate and compare the differences in GLS and MAPSE/AVPD between the modalities. Friedman’s test was used for analyzing repeated measurements of GLS and MAPSE/AVPD during chemotherapy. A statistical significance level of all analyses was set at a p-value < 0.05. All data are expressed as median values with interquartile range (IQR) unless otherwise stated.

## Results

A total of 16 participants were recruited and all of them were women. Various types of chemotherapeutic and cardiotoxic drugs were used in the treatment of these breast cancer patients, including EC (epirubicin and cyclophosphamide), Paclitaxel, Docetaxel Trastuzumab and Pertuzumab. Five of the participants did not fulfil the whole process of the study. Four of them withdrew after the baseline examination: one participant had their treatment changed and did not require chemotherapy, while the other three participants were not followed-up with echocardiography and were excluded automatically from the study. The fifth participant missed the follow-up control during cancer treatment but attended the final control after completing cancer treatment. Thus, 11 participants (69%) attended all controls throughout the study. All 11 of the participants went through three time points of examinations before they finished the study. Each participant remained in the study for a duration of approximately six months. A total of 39 ECHO and 39 CMR examinations were conducted between 2020 and 2022. The median time interval (IQR) between ECHO and the corresponding CMR was 0 days (0;4) and the median interval between follow-up assessments was 3 months (3;4).

At the baseline examination, the women (*n* = 16) included in the study had a median age (IQR) of 61 years (51;69.5). They had normal echocardiography with normal systolic and diastolic function (2D LVEF Simpson’s biplane 62 (59.7;67.6) %), normal-sized chambers (EDV 81.9 (67.2;108.9) ml and ESV 34.8 (24.9;52.2) ml), normal cardiac output (4.7 (3.8;6.3) l/ml) and insignificant valvular regurgitation. They had a heart rate of 76.5 (72;80) bpm during baseline ECHO and 81(66;91.3) bpm during baseline CMR. Table 1 summarizes the clinical, echocardiographic and CMR characteristics before, during and after chemotherapy on the participants (*n* = 11) that fulfilled the whole process of the study.

**Table 1:** Clinical, echocardiographic and CMR characteristics of the participants who fulfilled the whole study. All data are presented in median (IQR). Friedman’s test was used to compare each parameter during these three time sets

	Baseline	Control 1	Control 2	*P*-value
*n*	11	11	11	
*Clinical characteristics*				
Age (years)	60 (50;73)	60 (50;73)	60 (50;73)	
HR (ECHO) (bpm)	74 (72;78)	76 (72;83)	74 (63;81)	0.183
HR (CMR) (bpm)	81 (75;89)	81 (76;89)	71 (68;82)	**0.012**
*Echocardiographic characteristics*				
GLS QLab (%)	-20.4 (-20.7; -18.0)	-19.7 (-20.2; -18.2)	-19.2 (-19.8; -18.5)	0.148
GLS TomTec (%)	-22.0 (-22.7; -20.8)	-19.8 (-20.6; -19.4)	-20.1 (-20.9; -18.7)	**0.035**
Global MAPSE (mm)	16.8 (13.1;17.8)	16.5 (14.5;18.8)	15.5 (13.8;17.1)	0.076
2D LVEF bp (%)	64.3 (62.0;69.3)	64.9 (61.7;67.9)	62.8 (58.3;69.5)	0.529
EDV (ml)	81.3 (66.8;110.7)	82.8 (66.9;89.1)	75.0 (67.6;94.7)	0.695
ESV (ml)	27.1 (27.1;48.5)	27.6 (23.7;37.0)	29.4 (26.8;41.7)	0.441
CO (l/min)	4.3 (3.9;6.4)	4.8 (4.1;5.1)	4.9 (3.8;6.9)	0.559
*Magnetic resonance imaging characteristics*				
GLS (%)	-19.5 (-20.6; -17.4)	-17.9 (-19.5; -17.8)	-17.4 (-18.2; -17.0)	**0.004**
Global AVPD (mm)	14.0 (11.4;15.8)	13.0 (12.5;13.8)	13.8 (12.1;15.3)	0.706
LVEF (%)	71.0 (69.5;77.0)	72.0 (65.0;74.0)	65.0 (62.0;69.0)	**0.017**
EDV (ml)	116.0 (103.5;128.5)	130.0 (127.5;144.5)	123.0 (112.0;145.0)	**0.013**
ESV (ml)	32.0 (26.0;37.0)	40.0 (34.5;48.5)	40.0 (36.0;55.0)	0.079
CO (l/min)	7.5 (5.9;8.0)	6.8 (6.1;9.2)	5.7 (4.8;6.6)	**0.002**

HR, Heart rate; ECHO, echocardiography; CMR, cardiac magnetic resonance imaging; GLS, global longitudinal strain; MAPSE, mitral annular plane systolic excursion; AVPD, atrioventricular plane displacement; LVEF, left ventricle ejection fraction; bp, Simpson’s biplane; EDV, end-diastolic volume; ESV, end-systolic volume; CO, cardiac output

### ECHO versus CMR

Two different software programs, QLab and TomTEC, were used to obtain echocardiographic GLS values, resulting in two sets of measurements. Statistical analysis showed no significant differences in GLS values between ECHO and CMR (*n* = 16)(*p* = 0.733 for ECHO-GLS QLab vs. CMR and *p* = 0.093 for ECHO-GLS TomTec vs. CMR). However, ECHO-GLS QLab had a lower bias than ECHO-GLS TomTec compared to CMR-GLS (see additional file S5 and additional file S6).

A significant difference in global MAPSE and AVPD at baseline (*n* = 16) was observed (*p* = 0.015). Bland-Altman plot comparing the global mitral annular plane displacement between ECHO and CMR was also generated (see additional file S7).

### ECHO and CMR – before, during and after cancer treatment

**Fig. 2:**
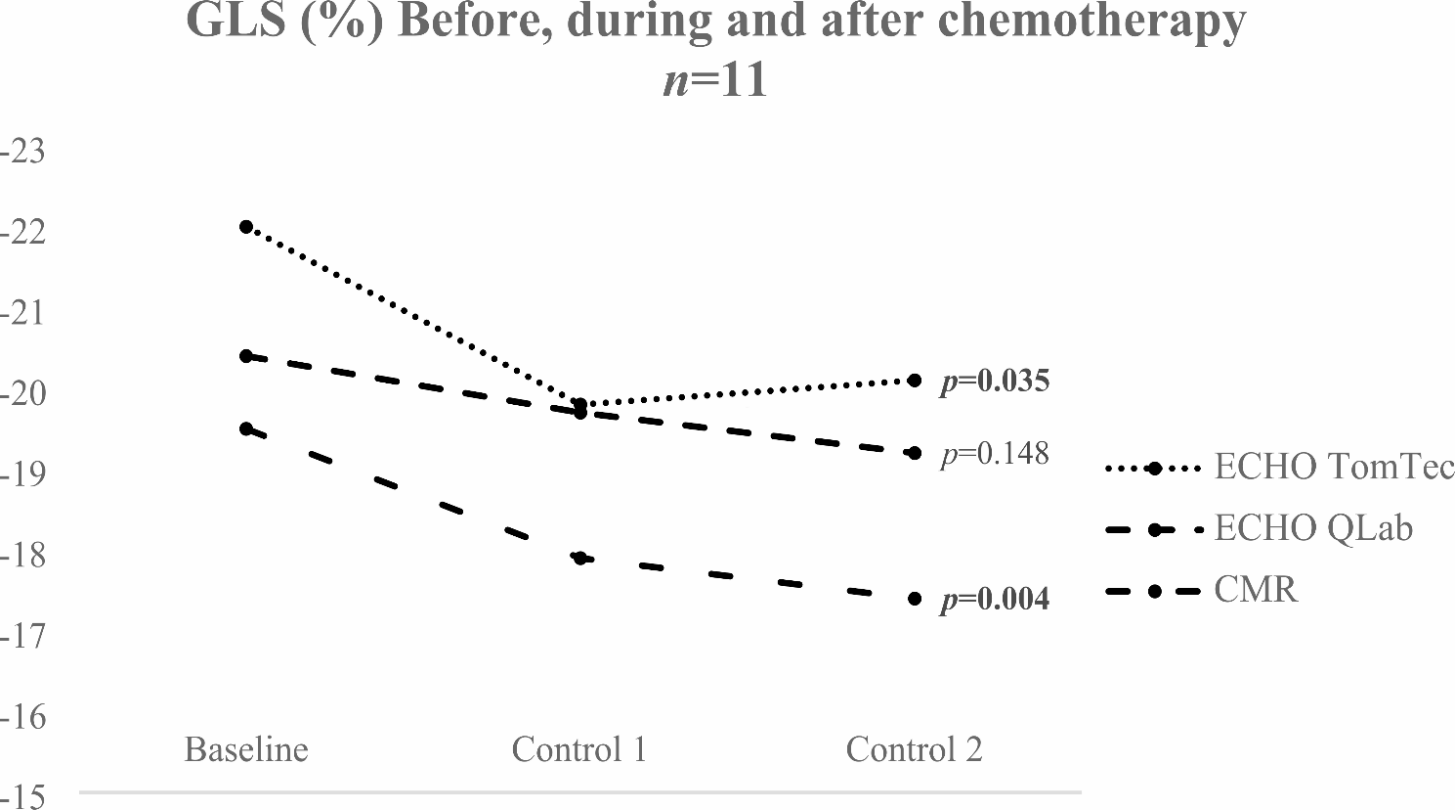
Median of GLS from ECHO and CMR before, during and after chemotherapy. Friedman’s test was used for the statistical analysis. ECHO, echocardiography; CMR, cardiac magnetic resonance; GLS, global longitudinal strain; (T), TomTec; (Q), QLab

**Fig. 3:**
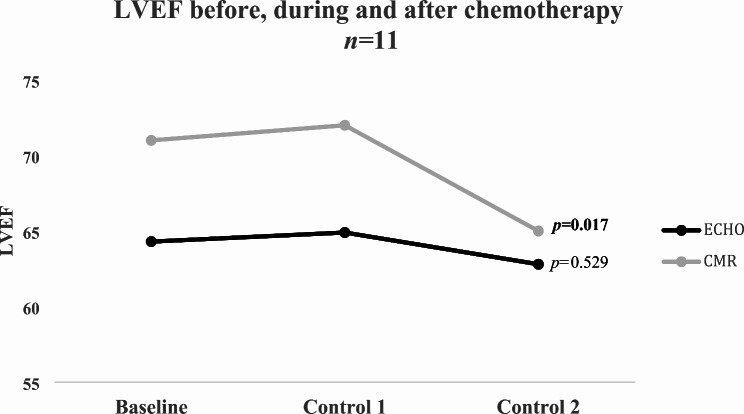
Median of LVEF before, during and after chemotherapy. Friedman’s test was used for the statistical analysis. LVEF, left ventricle ejection fraction; ECHO, echocardiography; CMR, cardiac magnetic resonance imaging

Repeated measurements of the myocardial deformation before, during and after cytostatic treatment was feasible in 11 out of 16 individuals (69%, *n* = 11). From the observation of the GLS values before, during and after chemotherapy ECHO TomTec and CMR showed significant differences after cytostatic treatment (*p* = 0.035 respective *p* = 0.004). However, GLS values obtained from ECHO QLab did not present statistical evidence of differences after cancer treatment (*p* = 0.148). Even so, Fig. 2 illustrates that all three trends indicated a marginal reduction in GLS after cytostatic treatment. Furthermore, LVEF obtained from CMR exhibited a significant decrease after treatment (*p* = 0.017), while 2D LVEF bp did not show a distinct decline (*p* = 0.529) (Fig. 3).

**Fig. 4:**
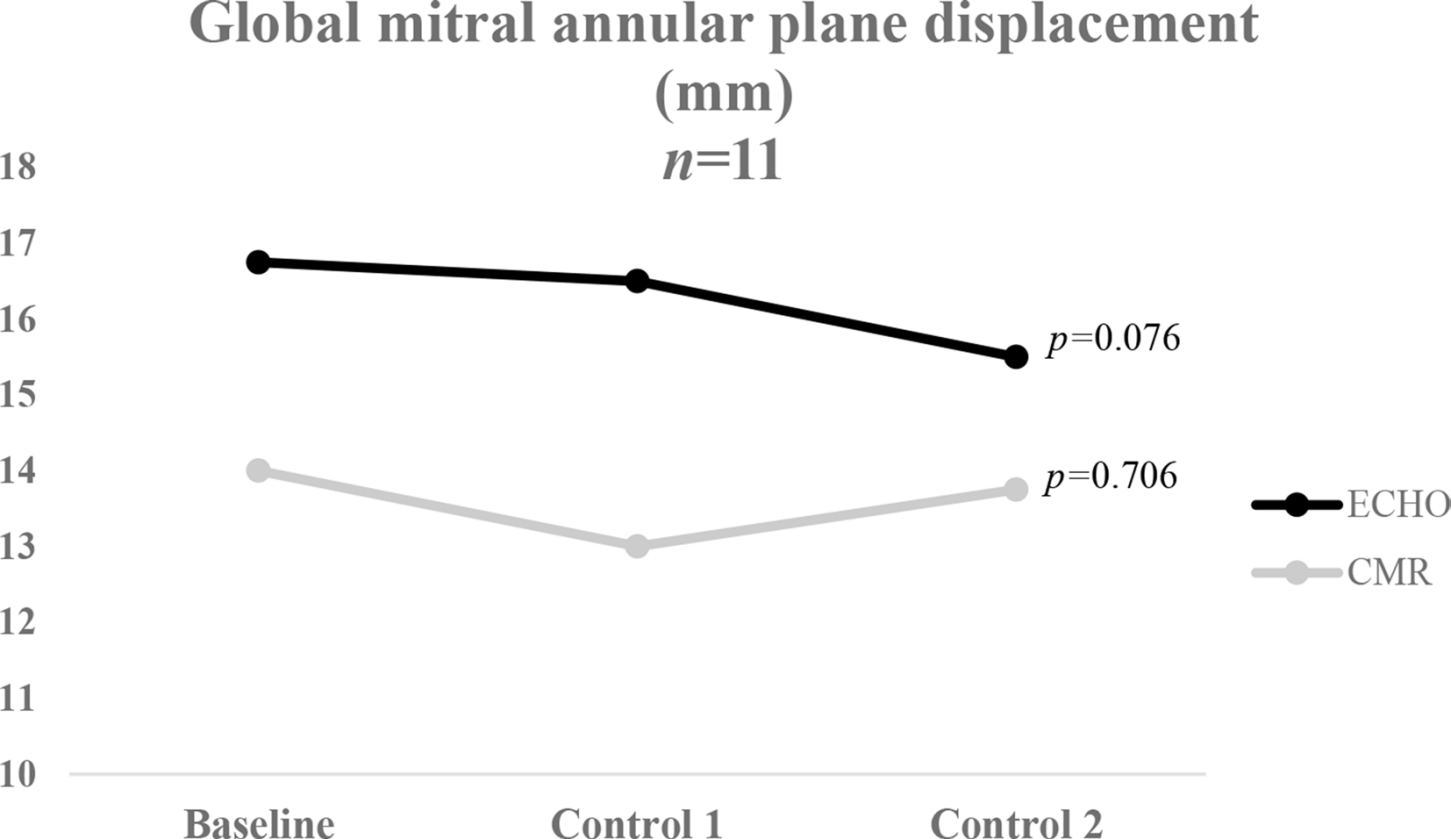
Median of the global mitral annular plane displacement obtained from ECHO and CMR before, during and after chemotherapy. Friedman’s test was used for the statistical analysis. ECHO, echocardiography; CMR, cardiac magnetic resonance imaging

Figure 4 demonstrates the comparison of the global mitral annular plane displacement (MAPSE respective AVPD) between ECHO and CMR before, during and after cancer treatment. There was no significant reduction in MAPSE or AVPD after the cancer treatment. However, the trend in ECHO-MAPSE values showed a reduction after chemotherapy, while CMR-AVPD did not decline after chemotherapy.

## Discussion

Detecting subtle myocardial changes enables clinicians to identify early signs of cardiac dysfunction, before appearance of heart failure symptoms. Early detection allows for timely interventions, such as adjusting the cancer treatment regimen or incorporating cardioprotective therapies, potentially preventing irreversible cardiac damage. This can improve long-term cardiac outcomes, as cardiovascular disease is a major cause of morbidity in cancer survivors, especially in patients with pre-existing cardiovascular risk factors. It also supports more personalized treatment strategies, optimizing cancer therapy while minimizing cardiac risk. Furthermore, validating that ECHO is as effective as CMR for detecting subtle myocardial changes has important clinical implications. By demonstrating that ECHO provides comparable diagnostic accuracy, clinicians can avoid the need of CMR in many cases. This is particularly beneficial since ECHO is more accessible and is less expensive. ECHO has also fewer challenges for patients, such as those with claustrophobia, metal implants or other contradictions to CMR.

There were no significant differences between the modalities ECHO and CMR regarding GLS. Additionally, the Bland-Altman plots (see additional file S1 and additional file S2) demonstrated that ECHO-GLS QLab compared with CMR-GLS had lower bias than ECHO-GLS TomTec compared to CMR-GLS. These variations could potentially be attributed to inter-vendor variabilities [15].

In Fig. 2, the trend is unanimous that GLS decreases after cytostatic treatment in all GLS measurements. However, only GLS obtained from ECHO TomTec and CMR presented significant differences when comparing GLS before, during and after chemotherapy. On the other hand, CMR-LVEF showed a significant decline after treatment, while ECHO-LVEF, which was obtained solely from a four- and two-chamber view, displayed a relatively unaltered trend (Fig. 3). Although no significant differences were observed between ECHO-GLS and CMR-GLS in this study, it is recommended to use consistent equipment and software for each individual to avoid any inter-vendor variations [16]. By doing so, it becomes more likely to detect subtle myocardial changes accurately.

The comparison between global MAPSE and AVPD indicates significant differences. Regarding MAPSE and AVPD before, during and after cytostatic treatment, as shown in Fig. 4, MAPSE had a small tendency to decrease following cancer treatment. CMR did not demonstrate a distinct reduction in AVPD. Furthermore, CMR generally displayed lower values than ECHO regarding GLS and mitral annular plane displacement, which could be explained by the FR discrepancy between the modalities.

GLS is considered a reliable measurement for monitoring the risk of CTRCD. Its ability to early identify CTRCD allows early adjustments of cardioprotective and chemotherapeutic treatments [17]. As mentioned earlier, longitudinal myocardial function is usually the first factor to be affected, underscoring the importance of evaluating longitudinal myocardial function to detect early stages of myocardial damage. In this study, there were variations in the outcomes of GLS and LVEF throughout the cancer treatment.

In an earlier study conducted in 2022 [18], a shared research focus emphasis with the present study was observed. In that study, the researches integrated the parameters GLS and myocardial work indices (MWI) to detect early signs of CTRCD. However, the current study included additional parameter, MAPSE/AVPD. Furthermore, compared two distinct imaging modalities, ECHO and CMR, and took into consideration the potential variations between different software programs for analysis.

Regrettably, this study had a small sample size. Moreover, the CMR software used could not assess the GLS of the endocardium (endo-GLS), which is usually the first layer to be affected in myocardial dysfunction [19, 20]. Endo-GLS may provide more sensitivity to detect subtle myocardial changes. Therefore, all GLS values in this study were based on an average GLS value of all layers of the myocardium. Another potential factor contributing to the variations between CMR and ECHO could be that these two examinations were not executed simultaneously. Most of them were examined with both modalities on the same day but some did the CMR many days (< 14 days) after their corresponding ECHO examination. A common limitation in echocardiography is suboptimal image quality due to poor acoustic windows, particularly in women, which compromises the accuracy of myocardial tracing. Some participants who underwent mastectomy during chemotherapy presented unhealed scars on their chests, making it more challenging to obtain high-quality images. We also wanted to keep this study focused and solely examine the modalities in the simplest way possible. While additional CMR data, such as tissue characterization by T1 or T2 mapping and biomarkers like NTproBNP and troponins, could have provided further insights, the primary goal was to compare the imaging modalities directly.

## Conclusion

ECHO-GLS and CMR-GLS are compatible with each other, as both methods were effective in detecting subtle reduction of the longitudinal myocardial function. Regarding mitral annular plane displacement, MAPSE demonstrated moderate sensitivity in detecting subtle myocardial changes.

## Electronic supplementary material

Below is the link to the electronic supplementary material.

Supplementary Material 1

## Data Availability

The data of the current study are available from the corresponding author on request.

## Abbreviations

ASE: American Society of Echocardiography
AVPD: Atrioventricular plane displacement
BP: Simpson’s biplane
CMR: Cardiac magnetic resonance imaging
CO: Cardiac output
CTRCD: Cancer therapy-related cardiac dysfunction
ECHO: Echocardiography
EDV: End-diastolic volume
Endo-GLS: Global longitudinal strain of the endocardium
ESV: End-systolic volume
FR: Frame rate
GLS: Global longitudinal strain
HR: Heart rate
IRQ: Interquartile range
ISCV: Intellispace cardiovascular
LVEF: Left ventricle ejection fraction
MAPSE: Mitral annular plane systolic excursion
MWI: Myocardial work indices
