# Supplementary material for: Comparison of echocardiographic and cardiac magnetic resonance imaging measurements of systolic function during breast cancer therapy
Source: BMC Cardiovasc Disord. 2025 Sep 15;25:653. doi: 10.1186/s12872-024-04262-7 (PMC12439410; doi:10.1186/s12872-024-04262-7)
Supplement: Supplementary file 1 — Supplementary Material 1 [file 12872_2024_4262_MOESM1_ESM.docx]

# Additional files


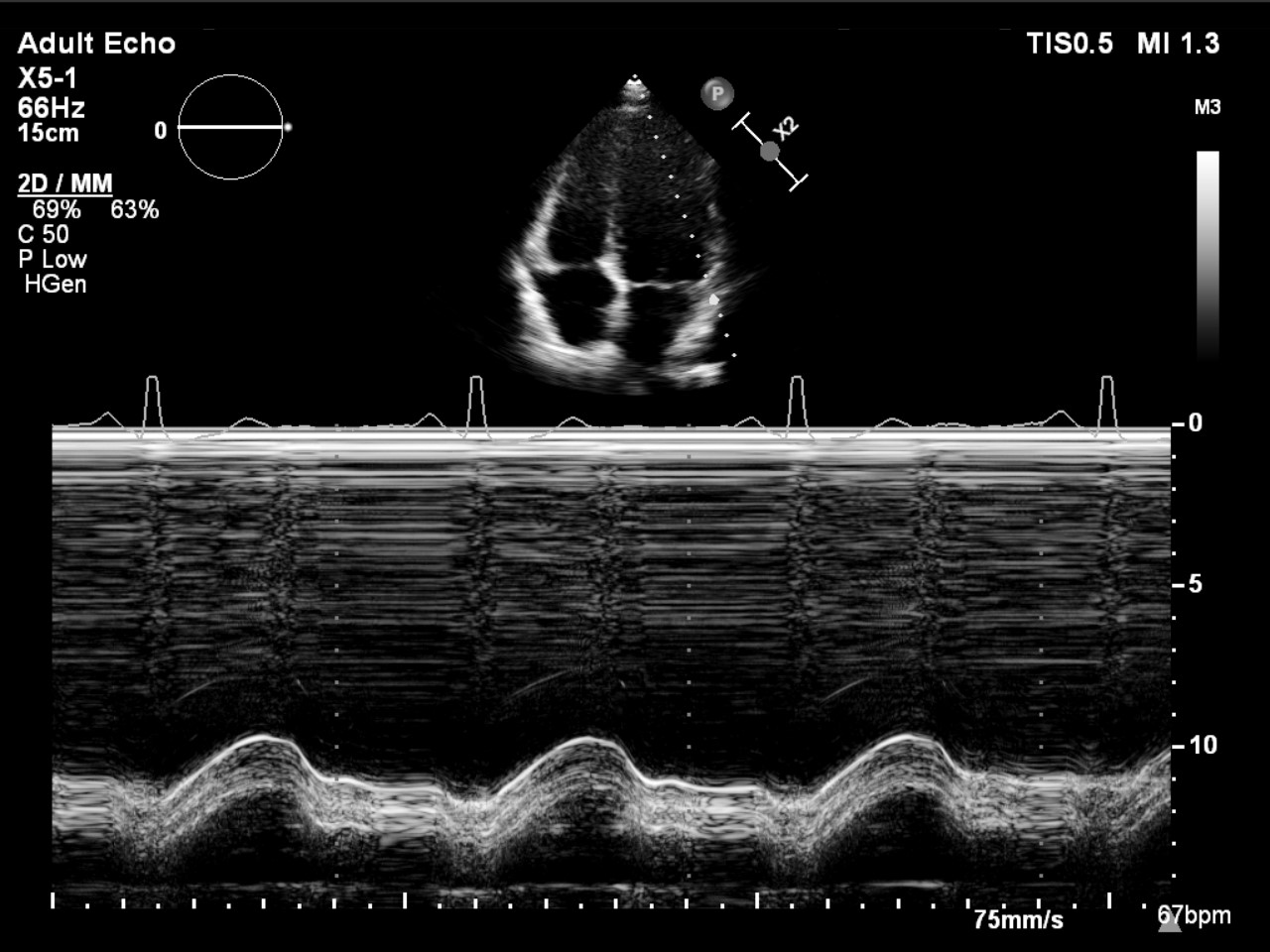


**Additional file 1.** MAPSE of the lateral left ventricle wall on four-chamber view using M-mode.

MAPSE, Mitral annular plane systolic excursion; M-mode, Motion-mode


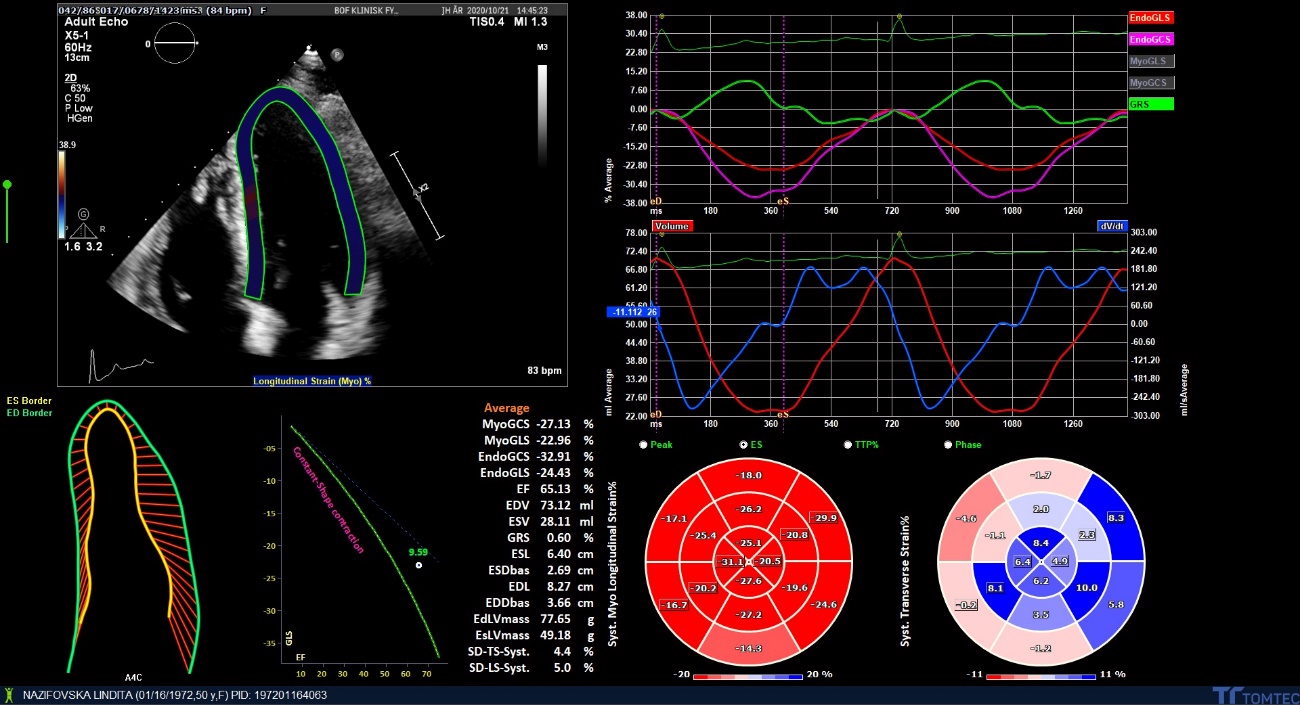


**Additional file 2.** ECHO GLS assessment with QLab on four-chamber view.

ECHO, Echocardiography; GLS, Global longitudinal strain.


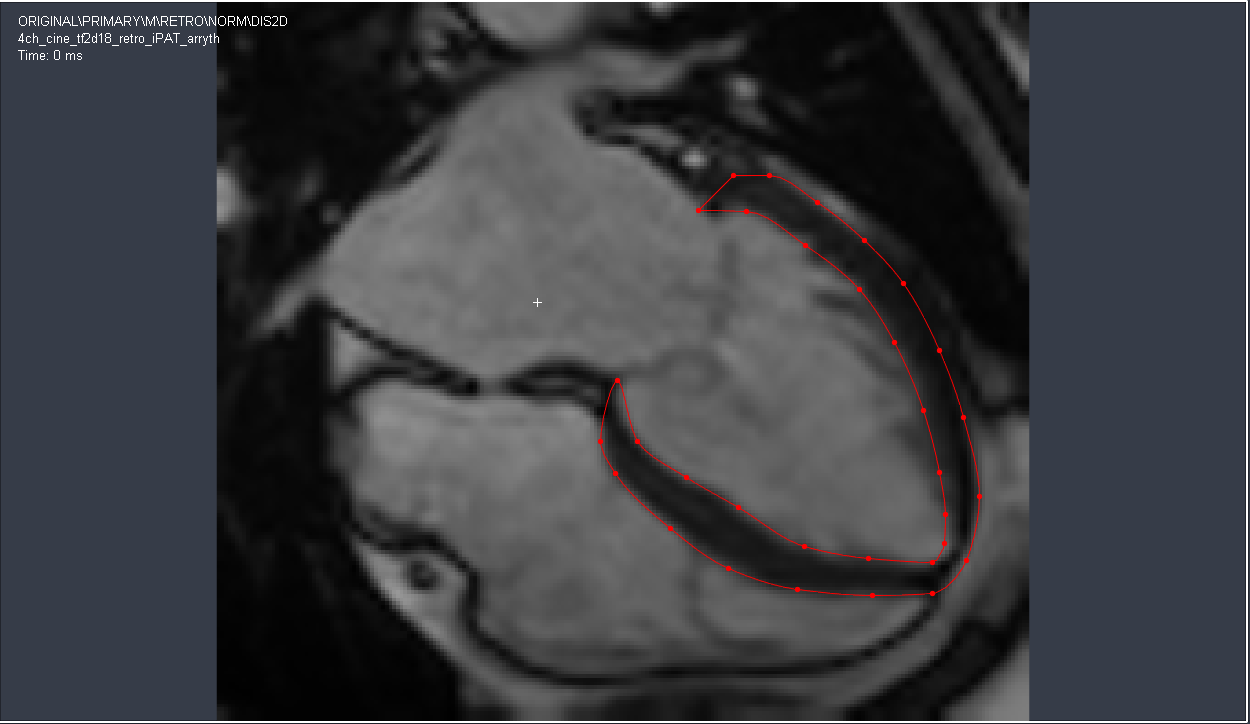


Additional file 3. CMR GLS assessment of the left ventricle in four-chamber view in analysis software Segment.

CMR, Cardiac magnetic resonance; GLS, Global longitudinal strain


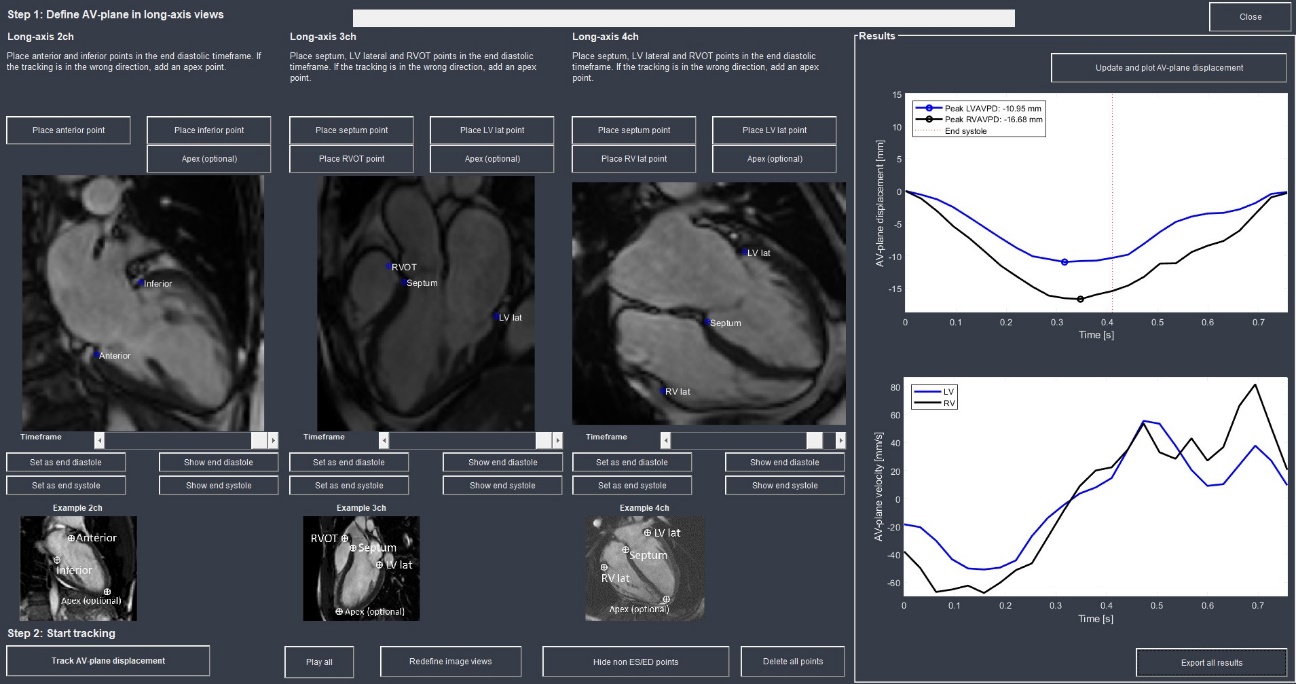


Additional file 4. AVPD assessment in analysis software Segment.

AVPD, Atrioventricular plane displacement


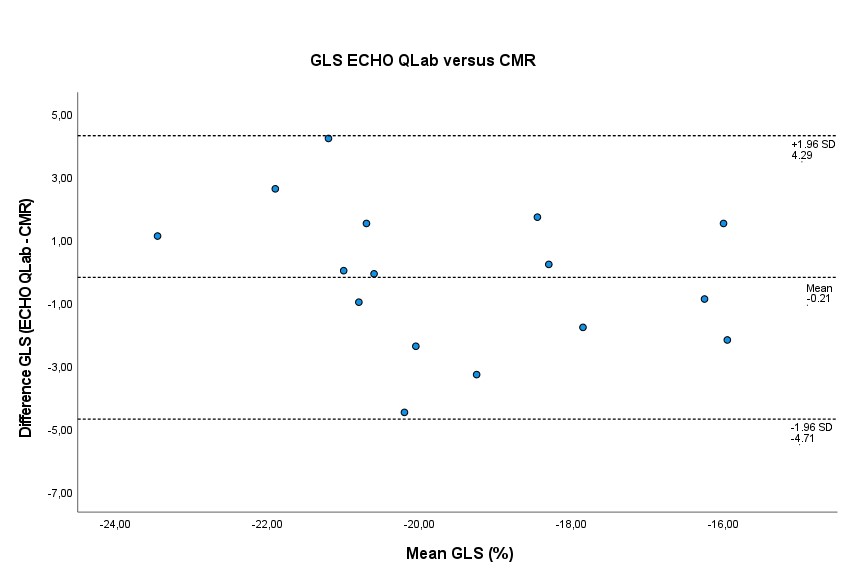


**Additional file 5.** A Bland-Altman plot of GLS between ECHO QLab and CMR.

GLS, global longitudinal strain; ECHO, echocardiography; CMR, cardiac magnetic resonance.


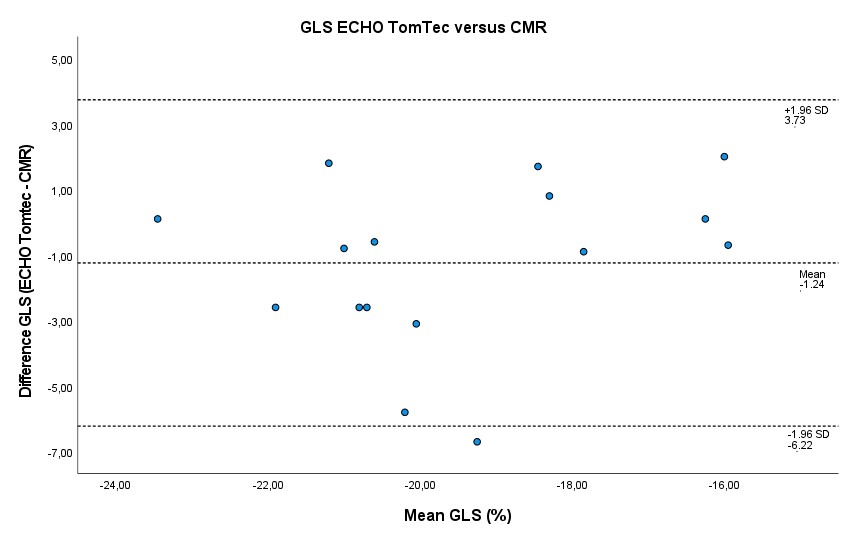


**Additional file 6.** A Bland-Altman plot of GLS between ECHO TomTec and CMR.

GLS, global longitudinal strain; ECHO, echocardiography; CMR, cardiac magnetic resonance


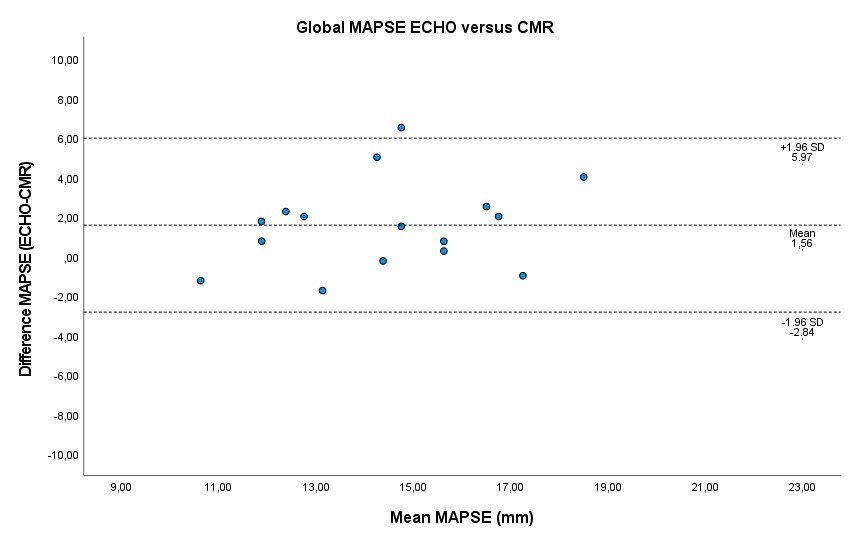


**Additional file 7.** A Bland-Altman plot of global mitral annular plane displacement between ECHO and CMR.

MAPSE, mitral annular plane systolic excursion; ECHO, echocardiography; CMR, cardiac magnetic resonance.
